# Global stability and bifurcations in a mathematical model for the waste plastic management in the ocean

**DOI:** 10.1038/s41598-024-71182-z

**Published:** 2024-09-02

**Authors:** Mahmood Parsamanesh, Mohammad Izadi

**Affiliations:** 1 Department of Mathematics, Technical and Vocational University, Tehran, Iran; 2 Department of Applied Mathematics, Shahid Bahonar University of Kerman, Kerman, Iran

**Keywords:** Waste plastic management, Compartmental model, Basic reproduction number, Bifurcation, Applied mathematics, Biological physics

## Abstract

The use of plastic is very widespread in the world and the spread of plastic waste has also reached the oceans. Observing marine debris is a serious threat to the management system of this pollution. Because it takes years to recycle the current wastes, while their amount increases every day. The importance of mathematical models for plastic waste management is that it provides a framework for understanding the dynamics of this waste in the ocean and helps to identify effective strategies for its management. A mathematical model consisting of three compartments plastic waste, marine debris, and recycle is studied in the form of a system of ordinary differential equations. After describing the formulation of the model, some properties of the model are given. Then the equilibria of the model and the basic reproduction number are obtained by the next generation matrix method. In addition, the global stability of the model are proved at the equilibria. The bifurcations of the model and sensitivity analysis are also used for better understanding of the dynamics of the model. Finally, the numerical simulations of discussed models are given and the model is examined in several aspects. It is proven that the solutions of the system are positive if initial values are positive. It is shown that there are two equilibria $E^0$ and $E^*$ and if ${{\mathcal {B}}}{{\mathcal {R}}}<1$, it is proven that $E^0$ is globally stable, while when ${{\mathcal {B}}}{{\mathcal {R}}}>1$, the equilibrium $E^*$ exists and it is globally stable. Also, at ${{\mathcal {B}}}{{\mathcal {R}}}=1$ the model exhibits a forward bifurcation. The sensitivity analysis of ${{\mathcal {B}}}{{\mathcal {R}}}$ concludes that the rates of waste to marine, new waste, and the recycle rate have most effect on the amount of marine debris.

## Introduction

The use of plastic is very widespread in the world and the spread of plastic waste has also reached the oceans. Observing marine debris is a serious threat to the management system of this pollution. Because it takes years to recycle the current wastes, while their amount increases every day. The importance of mathematical models for plastic waste management is that it provides a framework for understanding the dynamics of this waste in the ocean and helps to identify effective strategies for its management. In this paper, we study the asymptotic behavior of waste plastic process management (WPM) in the ocean using a three-compartment mathematical model consisting of plastic waste, marine debris, and recycled materials. This model can be described by the following nonlinear ordinary differential equation system^1^.

1 $$\begin{aligned} \begin{aligned} W'(t)&=\lambda -\gamma W(t)-\beta M(t) W(t)+\mu R(t),\\ M'(t)&=\beta M(t) W(t) -\alpha M(t),\\ R'(t)&=\gamma W(t)+\alpha M(t)-\left( \mu +\theta \right) R(t). \end{aligned} \end{aligned}$$

In this system, the time *t* (with a unit such as one day) is considered positive $(t>0)$ and the initial values of the variables are assumed to be non-negative values as follows $W(0)=W_{0}, M(0)=M_{0}, R(0)=R_{0}$. In this model, by *W*(*t*) we denote the amount of waste plastic material, *M*(*t*) is the marine debris, and *R*(*t*) represents the process of recycling. The descriptions of the coefficients of the WPM system are as follows. The parameter $\alpha$ is the marine debris rate to recycle and the waste rate to become marine denoted by $\beta$. Moreover, $\gamma$ is the waste rate to be recycled directly without utilizing marine debris and $\lambda$ describes the new waste rate to be reproduced. The parameter $\mu$ is the recycled waste rate to be reproduced as new waste and finally by $\theta$ we denote the recycled waste rate to be lost.

Using the properties of the Levenberg–Marquardt backpropagation (LBMBP), authors in^1^ designed an artificial neural network method for solving this model computationally. In^2^, the model was solved using two schemes based on a modification of the Morgan–Voyce (MV) functions by applying directly matrix collocation procedure and by using the quasi-linearization together with the modified MV collocation method. As mentioned, in previous studies, this model has been investigated numerically, but so far, its dynamics have not been comprehensively discussed in any work. In the present study, we intend to analyze this model with mathematical tools, although we also test the obtained results by simulation and numerical examples.

Compartmental models have been used in mathematical modeling to study the epidemics and ecological systems^3–6^. Also, there are some three-compartmental epidemic models to study diseases, including SIR or SIS models with vaccination or quarantine^7–11^, which may be useful for studying this model due to the nature of the cycle of propagation and recycling of plastic waste in the ocean. But according to our knowledge, the system studied in none of the works are completely compatible with the system related to this phenomenon in this paper.

In the present work, we first give some basic properties of the model in Section “The description of the model”. After showing that the solutions of the system are always non-negative, we obtain two equilibrium points for the system along with their existence conditions. Then, by applying the next generation method, we determine the basic reproduction number ${{\mathcal {B}}}{{\mathcal {R}}}$ for the model. The investigation of the long-term behavior of the model as a dynamical system is done through the analysis of the WPM system (1) stability at the equilibrium points in Section “Stability of the model”. Also, we state and prove the conditions in terms of this quantity under which the system is stable at each equilibrium point. The bifurcations of the model and the sensitivity analysis of the parameters are discussed in Sections “Bifurcations of the model” and “Sensitivity analysis”, respectively. Numerical experiences and simulation of the solutions of the system are also carried out via some examples in Section “Numerical experiments”. Finally, the results of the paper are summarized as conclusions.

## The description of the model

In this section we obtain some basic properties of the model such as the invariant set of solutions, the equilibria of the model and the basic reproduction number.

### Non-negativity of solutions

The following lemma states that the solutions of system (1) remain non-negative with positive initial values.

#### Lemma 1

*The set* $\Omega =\{(W, M, R): W\ge 0, M\ge 0, R\ge 0\}$ *is a positive invariant for system* (1).

#### Proof

Assume $W(0)>0$, $M(0)>0$ and $R(0)>0$. We let

$$\begin{aligned} \tau =\sup \{t>0 : W(t)\ge 0, M(t)\ge 0, ~ \text {and} ~ R(t)\ge 0\}, \end{aligned}$$

and so $\tau >0$ since all variables are continuously differentiable. Now, if $\tau =+\infty$ then the positivity of solutions holds, but if $0<\tau <+\infty$ then one of the variables is zero at $\tau$ and for $t>\tau$ is negative. Assume that $W(\tau )=0$ and $W(t)<0$ for $t>\tau$. From the differential equation corresponding to *W* we find

$$\begin{aligned} W'(\tau )=\lambda - \gamma W(\tau ) - \beta M(\tau ) W(\tau ) + \mu R(\tau ) = \lambda + \mu R(\tau ) \ge 0, \end{aligned}$$

Therefore *W* is non-decreasing at $\tau$ and $W(t)\ge 0$ for $t>\tau$. This is a contradiction with the previous assumption for $\tau <+\infty$ and thus for all *t* we have $W(t)\ge 0$. We can use a similar argument for variables *M* and *R* and conclude $M(0)\ge 0$ and $R(0)\ge 0$ for all $t>0$. Therefore the solutions of the system (1) are always non-negative and $\Omega =\{(W, M, R): W\ge 0, M\ge 0, R\ge 0\}$ is positive invariant for solutions of system (1). $\square$

### Equilibria of the model

Solving the following system of equations gives the equilibrium points (in the format of $({{\bar{M}}},{{\bar{W}}}, {{\bar{R}}})$) for model (1):

2 $$\begin{aligned} \begin{aligned}{}&\lambda -\gamma {{\bar{W}}} -\beta {{\bar{M}}} {{\bar{W}}} +\mu {{\bar{R}}}=0\\&\beta {{\bar{M}}} {{\bar{W}}} -\alpha {{\bar{M}}}=0\\&\gamma {{\bar{W}}} + \alpha {{\bar{M}}} -\left( \mu + \theta \right) {{\bar{R}}}=0. \end{aligned} \end{aligned}$$

Two equilibrium points are obtained; the equilibrium

$$\begin{aligned} E^0=(M^0,W^0,R^0)=\left( 0, \frac{ (\mu +\theta )\lambda }{\theta \gamma }, \frac{\lambda }{\theta }\right) \end{aligned}$$

if ${{\bar{M}}}=0$, and the equilibrium

$$\begin{aligned} E^*=(M^*,W^*,R^*)=\left( \frac{(\mu +\theta )\lambda }{\theta \alpha }-\frac{\gamma }{\beta },\frac{\alpha }{\beta } , \frac{\lambda }{\theta }\right) . \end{aligned}$$

when $${{\bar{M}}}>0$$.

#### Basic reproduction number

First, we note that $M^*$ in equilibrium point $E^*$ can be written as

$$\begin{aligned} M^*=\frac{(\mu +\theta )\lambda }{\theta \alpha }-\frac{\gamma }{\beta } =\frac{(\mu +\theta ) \lambda \beta - \gamma \alpha \theta }{\alpha \theta \beta }. \end{aligned}$$

Thus, the equilibrium point $E^*$ exists if and only if

$$\begin{aligned} M^*>0 \Leftrightarrow \frac{\gamma }{\beta }\Big (\frac{\beta \lambda (\mu +\theta )}{\gamma \alpha \theta }-1\Big )>0. \end{aligned}$$

Therefore if we let ${{\mathcal {B}}}{{\mathcal {R}}}=\frac{\beta \lambda (\mu +\theta )}{\alpha \gamma \theta }$, then $M^*=\frac{\gamma }{\beta }({{\mathcal {B}}}{{\mathcal {R}}}-1)>0$ if and only if ${{\mathcal {B}}}{{\mathcal {R}}}>1$ and thus $E^*$ exists if and only if ${{\mathcal {B}}}{{\mathcal {R}}}>1$. On the other hand, considering that the components of $E^0$ are always non-negative, we have stated the following lemma.

##### Lemma 2

*For model* (1)*, the equilibrium point* $E^0$ *always exists, while the equilibrium point* $E^*$ *exists under the condition that* ${{\mathcal {B}}}{{\mathcal {R}}}>1$.

We call ${{\mathcal {B}}}{{\mathcal {R}}}$ as the basic reproduction number of the WPM model. The basic reproduction number for a compartmental model is actually defined as the number of secondary reproductions in a completely homogeneous population by a member of a compartment that causes the spread of undesirable properties in other compartments^12^. In the preceding discussions, this quantity was implicitly given by using the condition that the amount of marine debris ($M^*$) is positive at the equilibrium point $E^*$. Moreover, it can be also obtained by the next generation matrix method as the spectral radius of the Jacobian matrix of the model at $E^0$ as follows^13^.

In this method we rewrite the second equation in (1) corresponding to the marine debris (compartment *M*) as

$$\begin{aligned} M'(t)=\beta M(t) W(t) -\alpha M(t) ={\mathcal {F}}-{\mathcal {V}}, \end{aligned}$$

where ${\mathcal {F}}=\beta MW$ and ${\mathcal {V}}=\alpha M$. Here, ${\mathcal {F}}$ refers to the terms in the equation that generate new amounts of marine debris (*M*), and ${\mathcal {V}}$ consists of terms that represent the transmissions between compartment *M* and other compartments.

Now supposing $f=\frac{\partial {\mathcal {F}}}{\partial {M}}({E^0})= \beta W^0 = \frac{\beta \lambda (\mu +\theta )}{\gamma \theta }$ and $v=\frac{\partial {\mathcal {V}}}{\partial {M}}({E^0})=\alpha$, then the basic reproduction number of the model is obtained by

3 $$\begin{aligned} {{\mathcal {B}}}{{\mathcal {R}}}=\rho (f v^{-1})= \frac{\beta \lambda (\mu +\theta )}{\alpha \gamma \theta }. \end{aligned}$$

## Stability of the model

In the paper^2^, the authors studied the local asymptotic stability of the model. By examining the eigenvalues of the Jacobian matrix at the equilibrium points of the model and using the Routh-Hurwitz criterion^14^, they determined the conditions under which the eigenvalues of the Jacobian matrix have a negative real part and showed that when ${{\mathcal {B}}}{{\mathcal {R}}}<1$, the system is stable at equilibrium point $E^0$ and if ${{\mathcal {B}}}{{\mathcal {R}}}>1$, this point is unstable and equilibrium point $E^*$ is stable.

Now, in the following we investigate the global stability of the model at the equilibria of the system (1).

### Theorem 1

*The equilibrium* $E^0$ *of model* (1) *is globally asymptotically stable when* ${{\mathcal {B}}}{{\mathcal {R}}}\le 1$.

### Proof

For this purpose, we use the basic concept of global stability that states under the given conditions, all solutions of the model converge to the equilibrium point $E^0$ starting from any point^15^.

From third equation in system (1) we have

$$\begin{aligned} \begin{aligned} R(t)&=e^{-(\mu +\theta )t}\left\{ \int _0^t \left( \gamma W(s) + \alpha M(s) \right) e^{(\mu +\theta )s} ds + R(0)\right\} \\&=R(0) e^{-(\mu +\theta )t} + \int _0^t \left( \gamma W(s) + \alpha M(s) \right) e^{-(\mu +\theta )(t-s)} ds. \end{aligned} \end{aligned}$$

The first equation in (1) implies $W'(t)\le \lambda -\gamma W(t)+\mu R(t)$ and thus we get

$$\begin{aligned} \begin{aligned} W(t)&\le e^{-\gamma t} \left\{ \int _0^t \left( \lambda + \mu R(s)\right) e^{\gamma s} ds +W(0)\right\} \\&=W(0) e^{-\gamma t} + \int _0^t \left( \lambda + \mu R(s)\right) e^{-\gamma (t-s)} ds\\&=W(0) e^{-\gamma t} +\lambda \int _0^t e^{-\gamma (t-s)} ds + \mu R(0) \int _0^t e^{-(\mu +\theta )} e^{-\gamma (t-s)} ds\\&\quad +\mu \gamma \int _0^t \left( \int _0^s W(v) e^{-(\mu +\theta )(s-v)} dv\right) e^{-\gamma (t-s)} ds\\&\quad +\mu \alpha \int _0^t \left( \int _0^s M(r) e^{-(\mu +\theta )(s-r)} dr\right) e^{-\gamma (t-s)} ds\\&= W(0) e^{-\gamma t} +\frac{\lambda }{\gamma } \left( 1-e^{-\gamma t}\right) -\frac{\mu R(0)}{\mu +\gamma -\theta } \left( e^{-(\mu +\theta )t}-e^{-\gamma t}\right) \\&\quad -\frac{\mu \gamma }{\mu +\gamma -\theta } \left( \int _0^t W(s) e^{-(\mu +\theta )(t-s)} ds-\int _0^t W(s) e^{-\gamma (t-s)} ds - e^{-\gamma t}\right) \\&\quad -\frac{\mu \alpha }{\mu +\gamma -\theta } \left( \int _0^t M(s) e^{-(\mu +\theta )(t-s)} ds-\int _0^t M(s) e^{-\gamma (t-s)} ds - e^{-\gamma t}\right) . \end{aligned} \end{aligned}$$

Therefore we have

$$\begin{aligned} \begin{aligned} W(t)&\le W(0) e^{-\gamma t} +\frac{\lambda }{\gamma } \left( 1-e^{-\gamma t}\right) -\frac{\mu R(0)}{\mu +\gamma -\theta } \left( e^{-(\mu +\theta )t}-e^{-\gamma t}\right) \\&\quad -\frac{\mu }{\mu +\gamma -\theta } \left\{ \int _0^t \left( \gamma W(s)+\alpha M(s)\right) e^{-(\mu +\theta )(t-s)} ds \right. \\&\quad \left. -\int _0^t \left( \gamma W(s)+\alpha M(s)\right) e^{-\gamma (t-s)} ds -(\gamma +\alpha ) e^{-\gamma t}\right\} . \end{aligned} \end{aligned}$$

Now, from the second equation in system (1) we obtain

$$\begin{aligned} \begin{aligned} M'(t)&=\beta M(t) W(t) -\alpha M(t) \\&\le \beta M \left\{ \frac{\lambda }{\gamma } +\left( W(0)-\frac{\lambda }{\gamma }+\frac{\mu R(0)}{\mu +\gamma -\theta }+\frac{\mu (\gamma +\alpha )}{\mu +\gamma -\theta }\right) e^{-\gamma t} \right. \\&\quad -\frac{\mu R(0)}{\mu +\gamma -\theta }e^{-(\mu +\theta )t} -\frac{\mu }{\mu +\gamma -\theta } \int _0^t \left( \gamma W(s)+\alpha M(s)\right) e^{-(\mu +\theta )(t-s)} ds\\&\quad \left. +\frac{\mu }{\mu +\gamma -\theta } \int _0^t \left( \gamma W(s)+\alpha M(s)\right) e^{-\gamma (t-s)} ds -\frac{\alpha }{\beta } \right\} . \end{aligned} \end{aligned}$$

We consider two cases; if $\mu +\gamma >\theta$, then

$$\begin{aligned} \begin{aligned} M'(t)&\le \beta M \left\{ \frac{\lambda }{\gamma } +\left( W(0)-\frac{\lambda }{\gamma }+\frac{\mu R(0)}{\mu +\gamma -\theta }+\frac{\mu (\gamma +\alpha )}{\mu +\gamma -\theta }\right) e^{-\gamma t} \right. \\&\quad \left. +\frac{\mu }{\mu +\gamma -\theta } \int _0^t \left( \gamma W(s)+\alpha M(s)\right) e^{-\gamma (t-s)} ds -\frac{\alpha }{\beta } \right\} , \end{aligned} \end{aligned}$$

and thus there exists $\tau _1>0$ such that for $t>\tau _1$

$$\begin{aligned} M'(t)\le \beta M \left\{ \frac{\lambda }{\gamma } -\frac{\alpha }{\beta } \right\} , \end{aligned}$$

since $\frac{\theta }{\mu +\theta }<1$ and ${{\mathcal {B}}}{{\mathcal {R}}}\le 1$ implies $\frac{\lambda }{\gamma } < \frac{\alpha }{\beta }$. Therefore $\mathop {\lim }\limits _{t\rightarrow \infty } M(t) =0$ and in the followig limiting system

4 $$\begin{aligned} \begin{aligned} W'(t)&=\lambda -\gamma W(t)+\mu R(t),\\ R'(t)&=\gamma W(t)-\left( \mu +\theta \right) R(t), \end{aligned} \end{aligned}$$

we find that $\mathop {\lim }\limits _{t\rightarrow \infty } W(t) =\frac{\lambda (\mu +\theta )}{\gamma \theta }=W^0$ and $\mathop {\lim }\limits _{t\rightarrow \infty } R(t) =\frac{\lambda }{\theta }=R^0$. Therefore all trajectories of (4) converge to $(W^0, R^0)$ and the equilibrium $E^0$ is globally asymptotically stable.

But, if $\mu +\gamma <\theta$ we have

$$\begin{aligned} \begin{aligned} M'(t)&\le \beta M \left\{ \frac{\lambda }{\gamma } +\left( W(0)-\frac{\lambda }{\gamma }+\frac{\mu R(0)}{\mu +\gamma -\theta }+\frac{\mu (\gamma +\alpha )}{\mu +\gamma -\theta }\right) e^{-\gamma t} \right. \\&\quad \left. -\frac{\mu }{\mu +\gamma -\theta } \int _0^t \left( \gamma W(s)+\alpha M(s)\right) e^{-(\mu +\theta )(t-s)} ds -\frac{\alpha }{\beta } \right\} , \end{aligned} \end{aligned}$$

and with similar arguments we find again $\mathop {\lim }\limits _{t\rightarrow \infty } M(t) =0$ and thus the desired result is obtained. $\square$

The next theorem states the conditions under which the equilibrium $E^*$ is globally stable. For this purpose we use the Lyapunov’s direct method^16^ which has also been employed by many authors^17–21^

### Theorem 2

*The equilibrium* $E^*$ *of model* (1) *is globally asymptotically stable if* ${{\mathcal {B}}}{{\mathcal {R}}}>1$ *and* $\alpha =\gamma$.

### Proof

Using combinations of composite quadratic and common quadratic terms^17^, we consider function $L:\left\{ (M, W, R)\in \Omega : M, W, R >0\right\} \rightarrow {\mathbb {R}}$ as follows

$$\begin{aligned} L(M, W, R)=\frac{\theta }{2}(R-R^*)^2+\frac{\alpha }{2} \left[ (W-W^*)+(M-M^*)+(R-R^*)\right] ^2. \end{aligned}$$

Function *L* is $C^1$ on the interior of defined domain set, and we also see $L\ge 0$, the equilibrium point $E^*$ is the global minimum of *L*, and moreover $L(E^*) = 0$. By differentiating *L* we get

$$\begin{aligned} \begin{aligned} \frac{dL}{dt}&=\theta (R-R^*)R' +\alpha \left[ ((W-W^*)+(M-M^*)+(R-R^*)\right] \left( W'+M'+R'\right) \\&=\theta (R-R^*)\left[ \gamma W +\alpha M -(\mu +\theta ) R\right] +\alpha \left[ (W-W^*)+(M-M^*)+(R-R^*)\right] (\lambda -\theta R) \end{aligned} \end{aligned}$$

From system (2) we have

$$\begin{aligned} \begin{aligned}{}&\gamma W^* + \alpha M^* -\left( \mu + \theta \right) R^*=0\\&\lambda = \theta R^*. \end{aligned} \end{aligned}$$

Thus we can write

$$\begin{aligned} \begin{aligned} \frac{dL}{dt}&= \theta (R-R^*)\left[ \gamma (W-W^*) +\alpha (M-M^*) -(\mu +\theta ) (R-R^*)\right] \\&\quad +\alpha \left[ (W-W^*)+(M-M^*)+(R-R^*)\right] \left( -\theta (R-R^*)\right) \\&=(\gamma \theta -\alpha \theta )(W-W^*)(R-R^*)-\left( (\mu +\theta )\theta +\alpha \theta \right) (R-R^*)^2, \end{aligned} \end{aligned}$$

and thus since $\gamma =\alpha$, we have

$$\begin{aligned} \frac{dL}{dt}=-\left( (\mu +\theta )\theta +\alpha \theta \right) (R-R^*)^2\le 0. \end{aligned}$$

Also, $\frac{dL}{dt}=0$ if and only if $R=R^*$ (which implies from (1) $M=M^*$ and $W=W^*$) and

$$\begin{aligned} \left\{ (M, W, R)\in \Omega : \frac{dL}{dt}=0 \right\} =\left\{ E^* \right\} . \end{aligned}$$

Therefore the equilibrium $E^*$ is globally asymptotically stable according to LaSalle’s invariant principle^16^. $\square$

## Bifurcations of the model

We now consider the behavior of the model when ${{\mathcal {B}}}{{\mathcal {R}}}=1$. For this purpose we choose $\beta$ as the bifurcation parameter and from ${{\mathcal {B}}}{{\mathcal {R}}}=1$ we find

$$\begin{aligned} \beta ^*=\frac{\alpha \gamma \theta }{\lambda (\mu +\theta )}. \end{aligned}$$

To identify the bifurcations that model (1) exhibits, we apply center manifold theory^22^. According to this method we need to carry out the following change of model variables. Let $x_1=W$, $x_2=M$ and $x_3=R$ by using vector $X=(x_1,x_2,x_3)^T$, the waste plastic management model can be rewritten in the form $X'=F(X)$ with $F=(f_1, f_2, f_3)^T$ as follows

5 $$\begin{aligned} \begin{aligned} x_1'&=\lambda -\gamma x_2-\beta x_1 x_2+\mu x_3,\\ x_2'&=\beta x_1 x_2 -\alpha x_2,\\ x_3'&=\gamma x_1+\alpha x_2-\left( \mu +\theta \right) x_3. \end{aligned} \end{aligned}$$

The jacobian matrix is obtained as

$$\begin{aligned} J= \left( \begin{array}{ccc} -\gamma -\beta x_2 & -\beta x_1 & \mu \\ \beta x_2 & \beta x_1 -\alpha & 0 \\ \gamma & \alpha & -(\mu + \theta ) \\ \end{array} \right) , \end{aligned}$$

and at equilibrium $E^0$ for $\beta *$ (when ${{\mathcal {B}}}{{\mathcal {R}}}=1$) is

$$\begin{aligned} J_{\beta ^*}^0=J(E^0, \beta ^*) = \left( \begin{array}{ccc} -\gamma & -\alpha & \mu \\ 0 & 0 & 0 \\ \gamma & \alpha & -(\mu + \theta ) \\ \end{array} \right) , \end{aligned}$$

because $\frac{\beta ^* \lambda (\mu +\theta )}{\gamma \theta }=\alpha$. The eigenvalues of $J_{\beta ^*}^0$ are $\lambda _1=0$ and those for the sub-matrix

6 $$\begin{aligned} \Psi =\left( \begin{array}{cc} -\gamma & \mu \\ \gamma & -(\mu + \theta ) \\ \end{array} \right) . \end{aligned}$$

For this matrix we obtain

$$\begin{aligned} trace(\Psi )=-(\mu +\theta +\gamma )<0 ~~ \text {and} ~~ det(\Psi )=\gamma (\mu +\theta )-\mu \gamma = \gamma \theta >0, \end{aligned}$$

and thus its eigenvalues have negative real part^14^.

The right eigenvector $u=\left( u_1, u_2, u_3\right) ^T$ of matrix $J_{\beta ^*}^0$ corresponding to the zero eigenvalue is obtained by solving the system $J_{\beta ^*}^0 u=0$ and we get $u_1=-\alpha$, $u_2=\gamma$ and $u_3=0$. Besides, the components of the left eigenvector $v=\left( v_1, v_2, v_3\right)$ corresponding to the zero eigenvalue can be found from $v\dot{u}=1$ as are gotten as $v_1=0$ and $v_2=\frac{1}{\gamma }$ ad $v_3=0$. It can easily be checked that $v J_{\beta ^*}^0=0$. According to the center manifold theory we have to calculate the two following coefficients

$$\begin{aligned} a = \sum \limits _{k,i,j = 1}^3 {{v_k}{u_i}{u_j}} \frac{{\partial ^2}{f_k}}{\partial {x_i}\partial {x_j}}, \quad b = \sum \limits _{k,i = 1}^3 {{v_k}{u_i}} \frac{{\partial ^2}{f_k}}{\partial {x_i}\partial {\beta }}. \end{aligned}$$

All second order partial derivatives of $f_k, (k=1, 2, 3)$ with respect to $x_i, (i=1, 2, 3)$ and $\beta$ are calculated at $(E^0, \beta ^*)$ with $E^0=(x_1^*, x_2^*, x_3^*)=\left( \frac{\lambda (\mu +\theta )}{\gamma \theta }, 0 , \frac{\lambda }{\theta }\right)$. Since $v_1$, $v_3$ and $u_3$ are zero, we only need to calculate the following second order partial derivatives:

$$\begin{aligned} \begin{aligned} \frac{{\partial ^2}{f_2}}{\partial {x_2}\partial {x_1}}(E^0, \beta ^*)&= \frac{\partial }{\partial x_2}\left( \beta x_2\right) \Big |_{(E^0, \beta ^*)} = \beta ^*,\\ \frac{{\partial ^2}{f_1}}{\partial {x_1}\partial {\beta }}(E^0, \beta ^*)&=\frac{\partial }{\partial x_1}\left( x_1 x_2\right) \Big |_{(E^0, \beta ^*)}= 0,\\ \frac{{\partial ^2}{f_1}}{\partial {x_2}\partial {\beta }}(E^0, \beta ^*)&=\frac{\partial }{\partial x_2}\left( x_1 x_2\right) \Big |_{(E^0, \beta ^*)}= \frac{\lambda (\mu +\theta )}{\gamma \theta }. \end{aligned} \end{aligned}$$

Thus

$$\begin{aligned} \begin{aligned}{}&a = {v_2}{u_1}{u_2} \frac{{\partial ^2}{f_1}}{\partial {x_1}\partial {x_2}}(E^0, \beta ^*) = -\alpha \beta ^*<0, \\&b = {v_2}{u_1} \frac{{\partial ^2}{f_1}}{\partial {x_1}\partial {\beta }} +{v_2}{u_2} \frac{{\partial ^2}{f_1}}{\partial {x_2}\partial {\beta }}= 0 + \frac{\lambda (\mu +\theta )}{\gamma \theta } >0. \end{aligned} \end{aligned}$$

Therefore, according to Theorem 4.1 in^22^ since $a<0$ and $b>0$ we conclude that there is a forward bifurcation (transcritical bifurcation) at $\beta ^*$ (when ${{\mathcal {B}}}{{\mathcal {R}}}=1$) for the waste plastic management model.

## Sensitivity analysis

To find out how sensitive a function is to changes in the variables in its formula, the method of sensitivity analysis is used. This method uses a quantity called the normalized forward sensitivity index as a measure of the sensitivity of each variable. Since the basic reproduction number has a significant impact on the behavior of the model, we calculate the sensitivity indices of ${{\mathcal {B}}}{{\mathcal {R}}}$ for its variables, which are $\beta$, $\lambda$, $\mu$, $\alpha$, $\gamma$, and $\theta$.

The normalized forward sensitivity index of variable ${{\mathcal {B}}}{{\mathcal {R}}}$ for a variable *v*, is defined by ${{\mathcal {S}}}{{\mathcal {I}}}_v^{{\mathcal {R}}_0}=\frac{v}{{{\mathcal {B}}}{{\mathcal {R}}}} \times \frac{\partial {{{\mathcal {B}}}{{\mathcal {R}}}}}{\partial v}$^23,24^.

If ${{\mathcal {S}}}{{\mathcal {I}}}_v^{{{\mathcal {B}}}{{\mathcal {R}}}}>0$ then the variable *v* has positive impact on ${{\mathcal {B}}}{{\mathcal {R}}}$ and the value of variable ${{{\mathcal {B}}}{{\mathcal {R}}}}$ increases by increasing the value of *v*. While ${{\mathcal {S}}}{{\mathcal {I}}}_v^{{{\mathcal {B}}}{{\mathcal {R}}}}<0$ shows the reverse impact of *v* on ${{\mathcal {B}}}{{\mathcal {R}}}$; increasing the value of *v* implies decreasing in the value of ${\mathcal {R}}_0$. Also, the magnitude of ${{\mathcal {S}}}{{\mathcal {I}}}_v^{{{\mathcal {B}}}{{\mathcal {R}}}}$ shows the proportion of changes in ${{\mathcal {B}}}{{\mathcal {R}}}$ with respect to *v*.

The normalized forward sensitivity indices for ${{\mathcal {B}}}{{\mathcal {R}}}$ are caculating as follows:

$$\begin{aligned} \begin{array}{ll} {{\mathcal {S}}}{{\mathcal {I}}}_\beta ^{{{\mathcal {B}}}{{\mathcal {R}}}}=1, & \quad {{\mathcal {S}}}{{\mathcal {I}}}_\lambda ^{{{\mathcal {B}}}{{\mathcal {R}}}}=1,\\ {{\mathcal {S}}}{{\mathcal {I}}}_\mu ^{{{\mathcal {B}}}{{\mathcal {R}}}}=\dfrac{\mu }{\mu +\theta }<1, & \quad {{\mathcal {S}}}{{\mathcal {I}}}_\alpha ^{{{\mathcal {B}}}{{\mathcal {R}}}}=-1,\\ {{\mathcal {S}}}{{\mathcal {I}}}_{\gamma }^{{{\mathcal {B}}}{{\mathcal {R}}}}=-1, & \quad {{\mathcal {S}}}{{\mathcal {I}}}_{\theta }^{{{\mathcal {B}}}{{\mathcal {R}}}}=-\dfrac{\mu }{\mu +\theta }<0. \end{array} \end{aligned}$$

We see that ${{\mathcal {S}}}{{\mathcal {I}}}_v^{{{\mathcal {B}}}{{\mathcal {R}}}}>0$ for $v=\beta , \lambda$ and $\mu$, but ${{\mathcal {S}}}{{\mathcal {I}}}_v^{{{\mathcal {B}}}{{\mathcal {R}}}}<0$ for $v=\alpha , \gamma$ and $\theta$. Thus, any increase (or decrease) in values of $\beta , \lambda$ and $\mu$ has direct impact on the value of ${\mathcal {R}}_0$. However, any increase (or decrease) in values of $v=\alpha , \gamma$ and $\theta$ has reverse impact on the value of ${{\mathcal {B}}}{{\mathcal {R}}}$.

## Numerical experiments

**Fig. 1:**
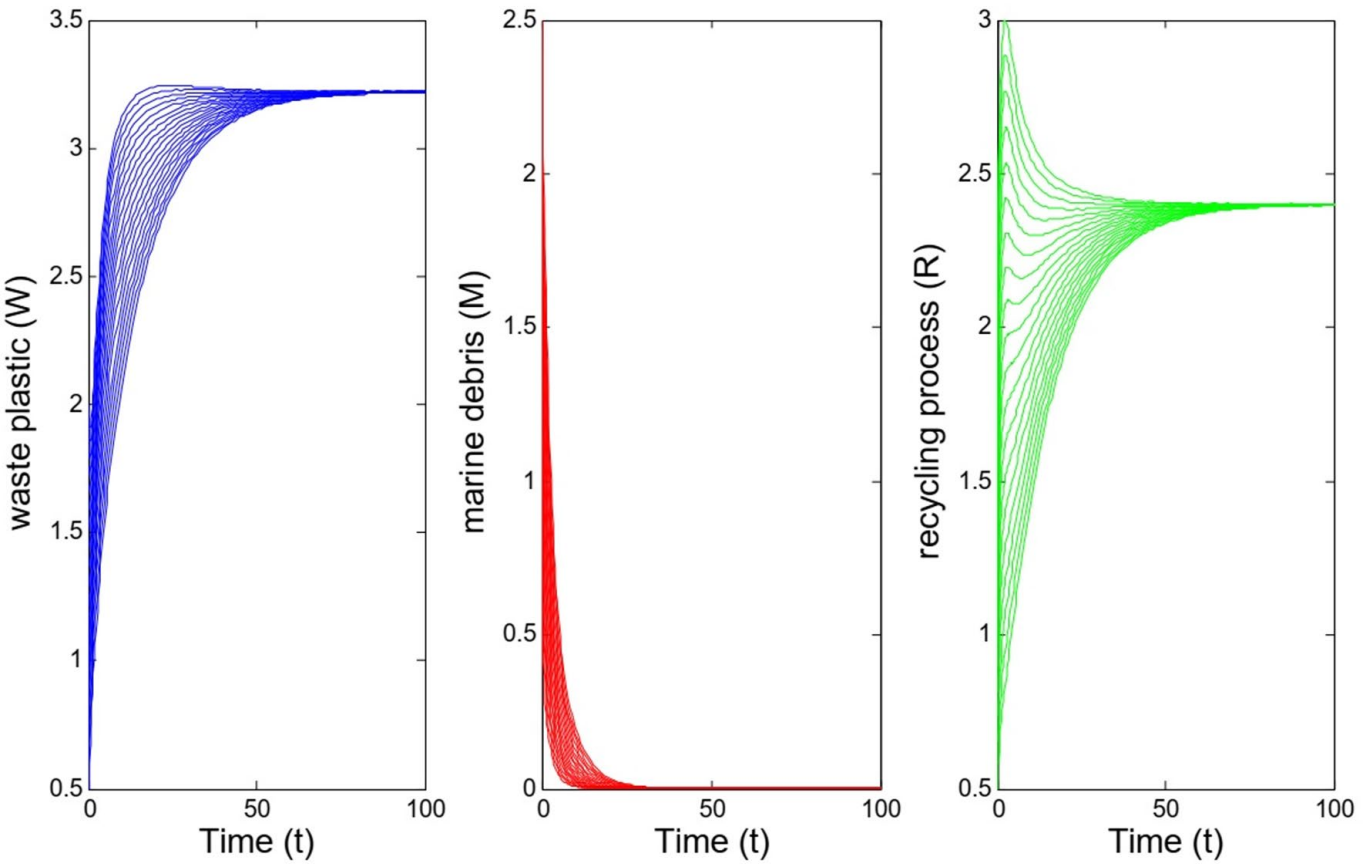
Solutions of model for different initial values for sub-populations. The parameter values are $\beta =0.15, \gamma =0.41, \alpha =0.65, \mu =0.4, \lambda =0.36, \theta =0.15$ which imply ${{\mathcal {B}}}{{\mathcal {R}}}=0.743<1$.

We consider the values $\beta =0.15, \gamma =0.41, \alpha =0.65, \mu =0.4, \lambda =0.36, \theta =0.15$ for the parameters in model (1). For these values we have ${{\mathcal {B}}}{{\mathcal {R}}}=0.743<1$ and according to Theorem 1 the equilibrium $E^0=(0, 3.2195, 2.4000)$ is stable. Figure 1 illustrates the solutions of the model with these parameter values and 20 different initial values for sub-populations. Moreover, the left picture in Fig. 3 shows the solutions of the system with initial values as $W_0=1.5$, $M_0=2$, and $R_0=1$.

**Fig. 2:**
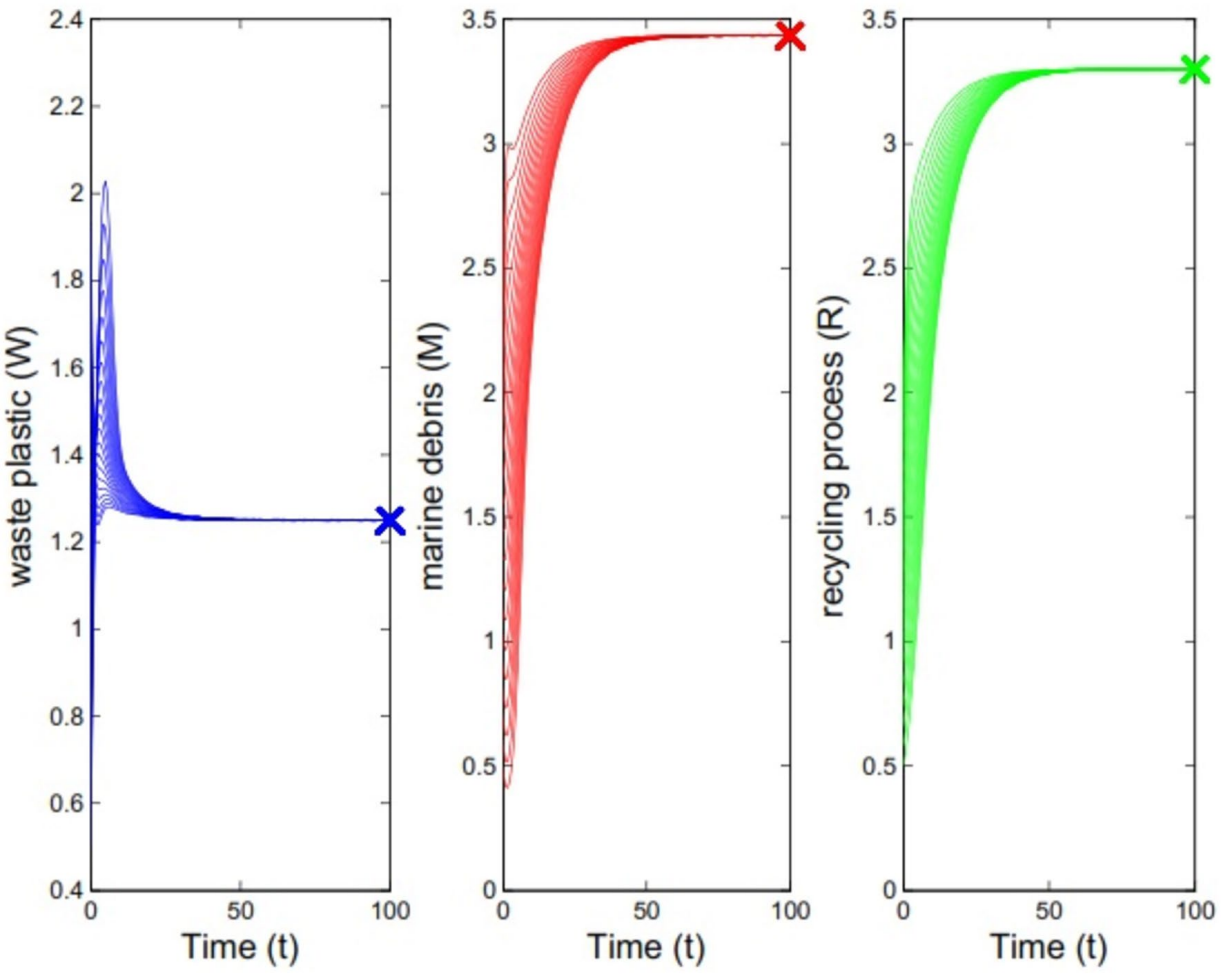
Solutions of model for different initial values for sub-populations. The parameter values are $\beta =0.4, \gamma =0.21, \alpha =0.5, \mu =0.4, \lambda =0.66, \theta =0.2$ which imply ${{\mathcal {B}}}{{\mathcal {R}}}=7.5429>1$.

**Fig. 3:**
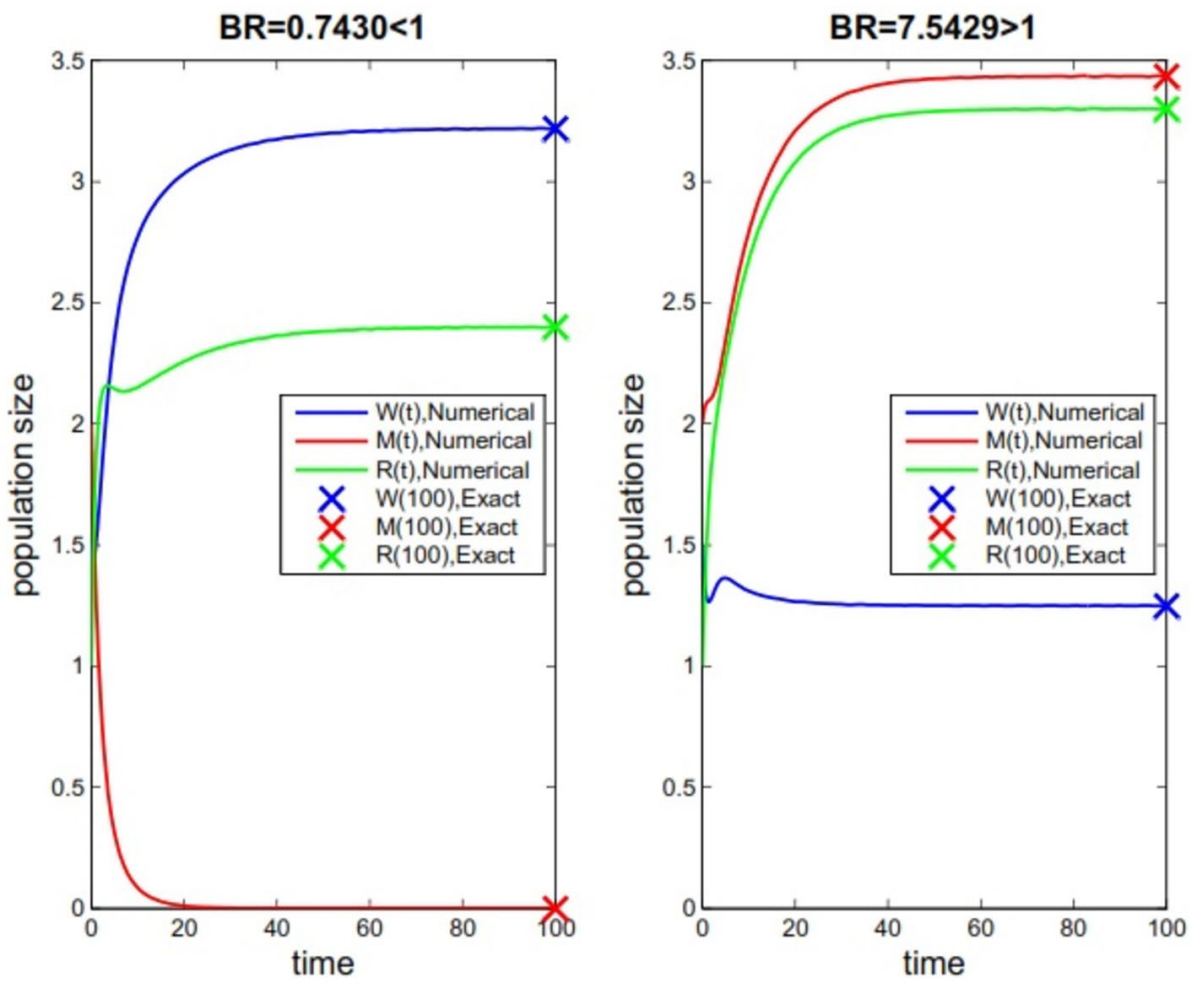
Solutions of the model for two cases ${{\mathcal {B}}}{{\mathcal {R}}}<1$ (the left picture) and ${{\mathcal {B}}}{{\mathcal {R}}}>1$ (the right picture) with their theoretical solutions when initial values are same.

Now, we change the parameter values to $\beta =0.4, \gamma =0.21, \alpha =0.5, \mu =0.4, \lambda =0.66, \theta =0.2$. Here, we have ${{\mathcal {B}}}{{\mathcal {R}}}=7.5429>1$ and by Theorem 2 the equilibrium $E^*=(1.25, 3.44, 3.30)$ is stable. In Fig. 2 the solutions of the system (1) has been depicted for 20 initial values for sub-populations. In the case of that the initial values are supposed as $W_0=1.5$, $M_0=2$, and $R_0=1$, the right picture in Fig. 3 shows the solutions of the model.

**Fig. 4:**
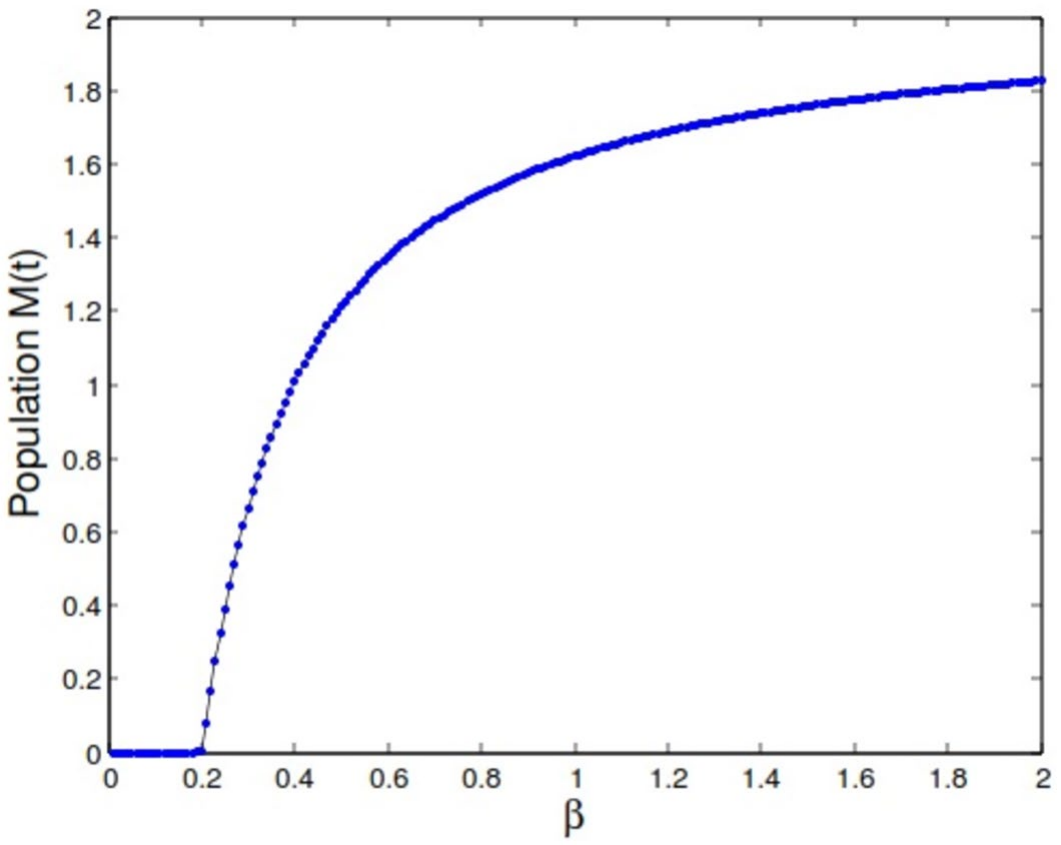
Bifurcation diagram of the model for parameter $\beta \in [0,2]$.

For parameter values $\gamma =0.41, \alpha =0.65, \mu =0.4, \lambda =0.36, \theta =0.15$ (as in the first example), if we solve the equation ${{\mathcal {B}}}{{\mathcal {R}}}=1$ for parameter $\beta$ as the bifurcation parameter, we get $\beta ^*=0.2019$. Thus for this value the model exhibit a forward bifurcation as it can be seen in bifurcation diagram presented in Fig. 4.

Now, we investigate the impact of the parameters of the model on the dynamics of the waste management system by using sensitivity analysis of the basic reproduction number ${{\mathcal {B}}}{{\mathcal {R}}}$ with respect to each parameter as it was explained in Section “Sensitivity analysis”. Let us consider the parameter values in Table 1 for parameters in the model introduced by system (1) and their corresponding sensitivity indices with respect to ${{\mathcal {B}}}{{\mathcal {R}}}$ as a differentiable function.

**Table 1:** Parameters in the waste management system (1) and their sensitivity index.

Parameter	Value	Sensitivity Index
$\beta$	0.75	+1
$\lambda$	0.66	+1
$\mu$	0.4	+0.8889
$\alpha$	0.5	$-1$
$\gamma$	0.21	$-1$
$\theta$	0.05	$-0.8889$

**Fig. 5:**
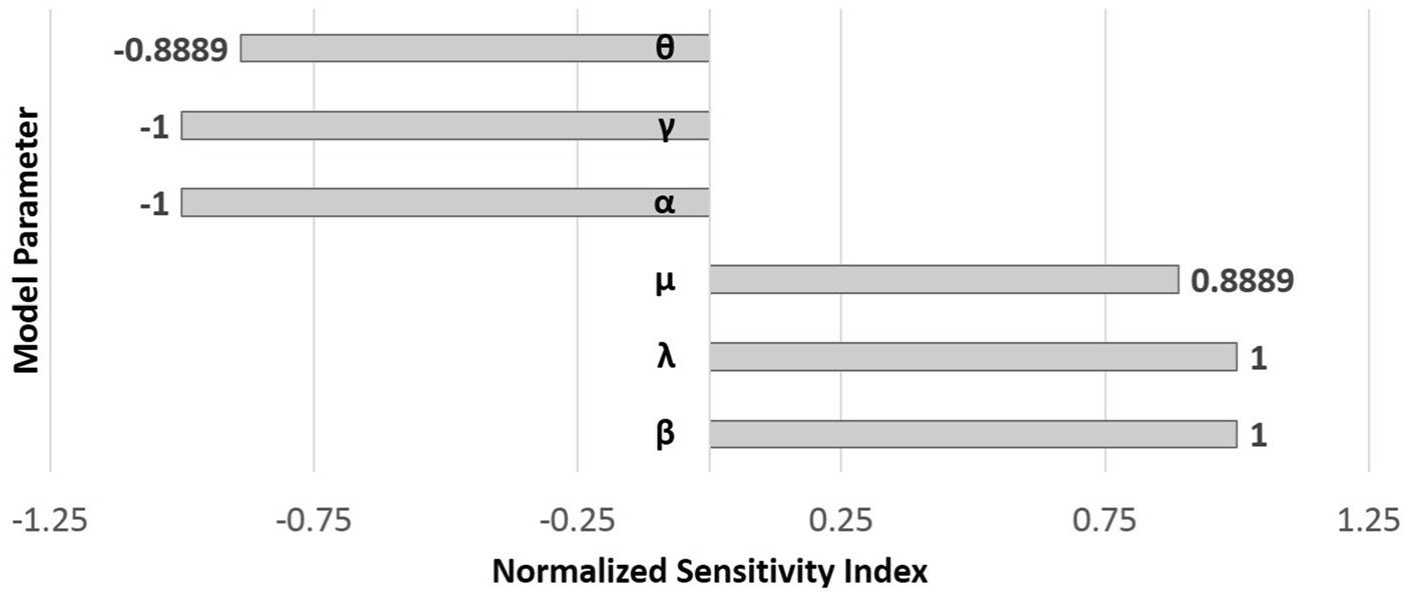
Normalized sensitivity indices for model parameters.

**Fig. 6:**
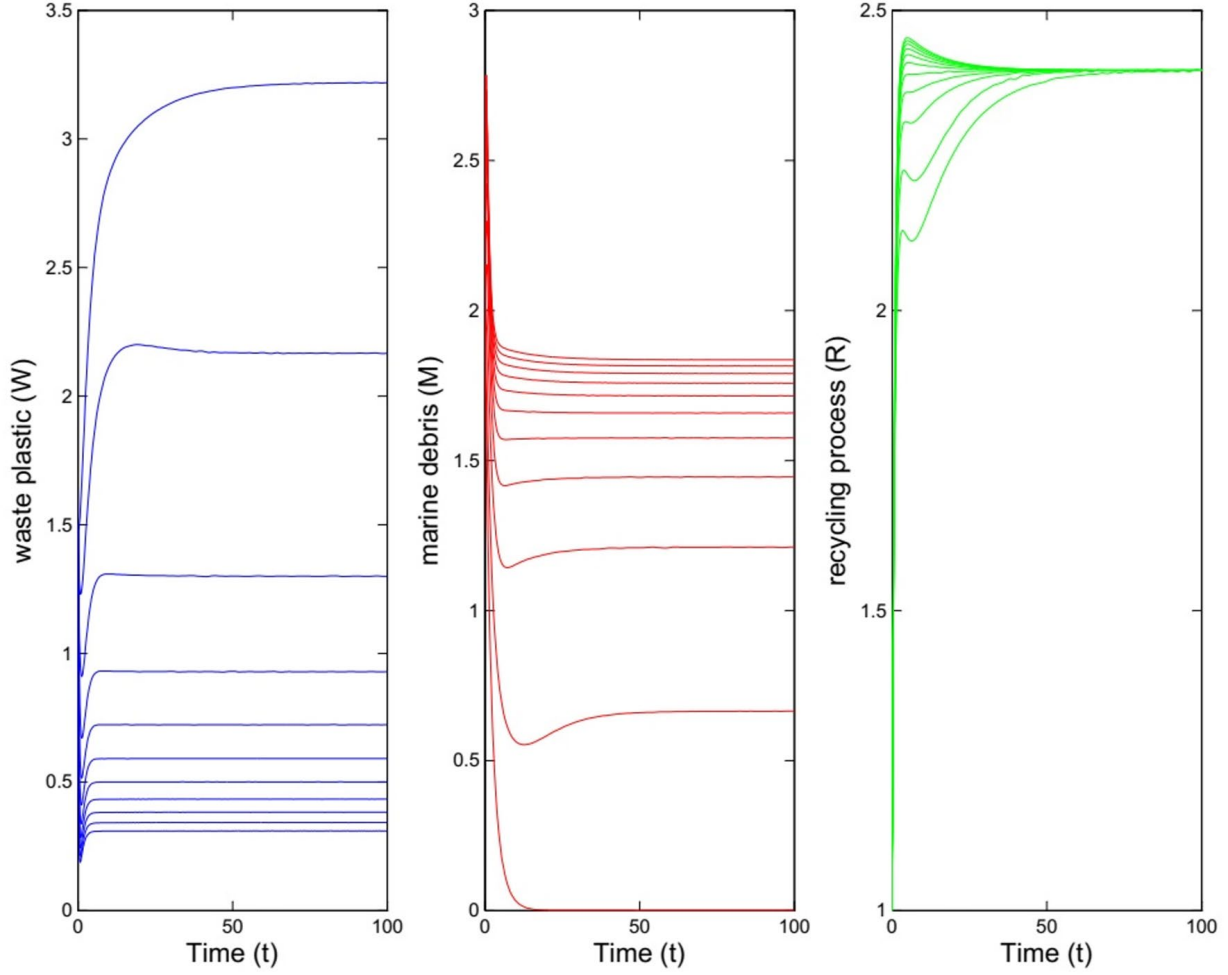
Solutions of the model for $\gamma =0.21, \alpha =0.5, \mu =0.4, \lambda =0.66, \theta =0.05$ and $\beta \in [0.1,2.1]$.

**Fig. 7:**
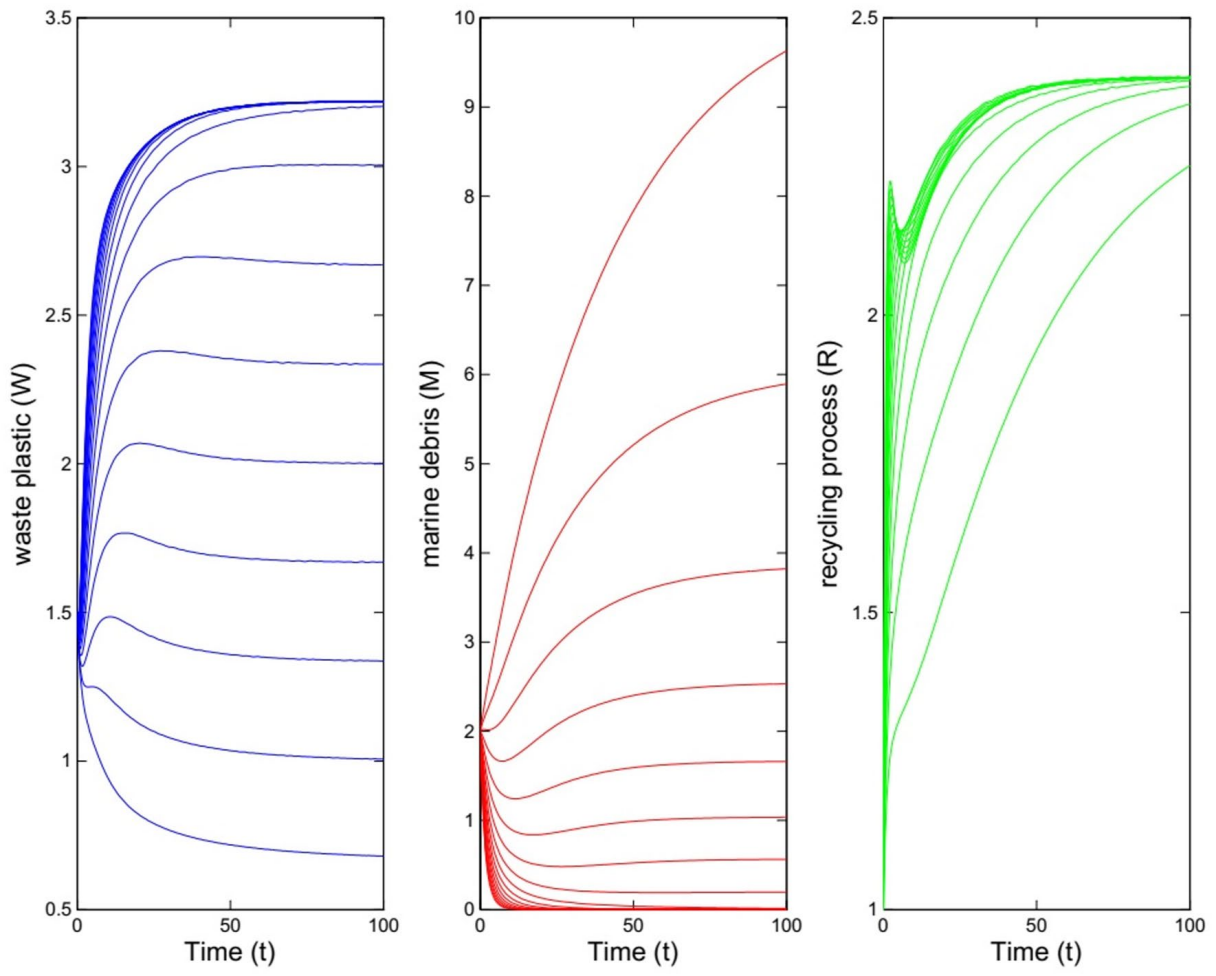
Solutions of the model for $\beta =0.75, \gamma =0.21, \mu =0.4, \lambda =0.66, \theta =0.05$ and $\alpha \in [0.1 , 0.9]$.

The normalized sensitivity indices for parameters have been shown as a chart in Fig. 5. According to the Table 1 we find that the sensitivity indices for parameters $\beta$, $\lambda$ and $\mu$ have positive value and their impact on ${{\mathcal {B}}}{{\mathcal {R}}}$ is direct. While, the parameters $\alpha$, $\gamma$ and $\theta$ have negative sensitivity indices and thus they have reverse impact on ${{\mathcal {B}}}{{\mathcal {R}}}$. Thus for example, an increase (decrease) in values of $\lambda$ and $\mu$ by %10 yields to a %10 and %8.889 increase (decrease) in ${{\mathcal {B}}}{{\mathcal {R}}}$, respectively. For instance, for parameter values in Table 1 we have ${{\mathcal {B}}}{{\mathcal {R}}}=42.4286$ and if we decrease $\lambda$ by %10, the basic reproduction number also decreases by %10 and becomes ${{\mathcal {B}}}{{\mathcal {R}}}=38.1857$. On the other hands, a %10 increase (decrease) for example in $\gamma$ and $\theta$ causes a %10 and %8.889 decrease (increase) in ${{\mathcal {B}}}{{\mathcal {R}}}$. For example, if the value of $\theta$ is increased by %20 (to $\theta =0.06$), then the value of ${{\mathcal {B}}}{{\mathcal {R}}}$ decreases by %17.778 and becomes ${{\mathcal {B}}}{{\mathcal {R}}}=34.8763$. Therefore, according to Table 1 we find that either decreasing the rates of waste to marine ($\beta$) and new waste ($\lambda$) or increasing the recycle rate ($\alpha$ and $\gamma$), have the most impact in reducing the value of ${{\mathcal {B}}}{{\mathcal {R}}}$ and as a result most impact on controlling the amount of marine debris. The impact of parameters $\beta$ and $\alpha$ on the model have also been depicted in Figs. 6 and 7, respectively. The parameter values are assumed as $\beta =0.75, \gamma =0.21, \alpha =0.5, \mu =0.4, \lambda =0.66, \theta =0.05$ and initial values are $W_0=1.5$, $M_0=2$, and $R_0=1$. In Fig. 6 the values of $\beta$ change in interval [0.1, 2.1] and it is seen that by increasing $\beta$ ,the final solution corresponding to the amount of the marine debris (*M*) leaves the zero and will take positive values. Indeed, the stability of the model changes from $E^0$ to $E^*$. With the same terms and for values of $\alpha$ in [0.1, 0.9], the solutions of the model have been shown in Fig. 7. By increasing the value of $\alpha$, we observe that the values of *M* finally take zero value. This shows that by increasing $\alpha$ the equilibrium point $E^*$ becomes unstable and $E^0$ becomes $E^*$ stable.

## Conclusions

In this paper, we studied the waste plastic management (WPM) system in the ocean through a mathematical three-compartmental model. The basic reproduction number ${{\mathcal {B}}}{{\mathcal {R}}}$, and two equilibria of the model were found, in addition to positivity of solutions of model. The dynamics of the model was determined in terms of threshold ${{\mathcal {B}}}{{\mathcal {R}}}$; if ${{\mathcal {B}}}{{\mathcal {R}}}<1$, it was proved that the equilibrium $E^0$ is globally stable, while the equilibrium $E^*$ exists and it is stable when ${{\mathcal {B}}}{{\mathcal {R}}}>1$. Also, it was shown that the model exhibit a forward (transcritical) bifurcation at ${{\mathcal {B}}}{{\mathcal {R}}}=1$. The sensitivity of the model has been analyzed by calculating normalized forward sensitivity index for each parameter for ${{\mathcal {B}}}{{\mathcal {R}}}$ and it was concluded that decreasing the rates of waste to marine ($\beta$) and new waste ($\lambda$) or increasing the recycle rate ($\alpha$ and $\gamma$), are most effective for controlling the amount of marine debris. Finally, the theoretical results were discussed also numerically for different parameter values and various initial values for sub-populations via several examples, solutions of model and bifurcation diagram.

The global stability of the equilibrium point $E^*$ has been proved for the case that the marine debris recycling rate ($\alpha$) and the direct recycling rate of waste materials ($\gamma$) are equal. Constructing a more appropriate Lyapunov function that does not impose such an additional condition on stability can be the subject of future studies. Investigating the impact of seasonality on the system behavior may also complement the present study, since the waste rate to become marine ($\beta$) can be considered as a periodic function.

## Data Availability

All data generated or analyzed during this study are included in this article.
